# Favipiravir, an antiviral drug, in combination with tamoxifen exerts synergistic effect in tamoxifen-resistant breast cancer cells via hTERT inhibition

**DOI:** 10.1038/s41598-024-51977-w

**Published:** 2024-01-22

**Authors:** Sally A. Fahim, Yehia A. ElZohairy, Rehab I. Moustafa

**Affiliations:** 1 Department of Biochemistry, School of Pharmacy, Newgiza University (NGU), Newgiza, Km 22 Cairo-Alexandria Desert Road, 6th of October, P.O. Box 12577, Giza, Egypt; 2 School of Pharmacy, Newgiza University (NGU), Newgiza, Km 22 Cairo-Alexandria Desert Road, P.O. Box 12577, Giza, Egypt; 3 Microbial Biotechnology Department, Biotechnology Research Institute, National Research Centre, Dokki, Giza, Egypt; 4 Microbiology Department, School of Pharmacy, Newgiza University (NGU), Newgiza, Km 22 Cairo-Alexandria Desert Road, P.O. Box 12577, Giza, Egypt

**Keywords:** Breast cancer, Transcription

## Abstract

Tamoxifen (TAM) is one of the most successful treatments for breast cancer; however, TAM resistance continues to be a significant barrier. TAM resistance has been reported to be associated with increased expression of human telomerase reverse transcriptase (hTERT). This enzyme shares structural similarity with RNA-dependent RNA polymerase (RdRp) enzyme of RNA viruses, suggesting that RdRp inhibitors may also inhibit hTERT. Favipiravir (FAV) is an antiviral drug that inhibits RdRp of RNA viruses. Thus, we propose that FAV may also elicit an antitumor effect by suppressing hTERT. This study aimed to investigate the effect of FAV and TAM on TAM-resistant breast cancer (TAMR-1). The cell viabilities were determined. The levels of CDK1/ hTERT, in addition to regulators of hTERT-targeted signaling pathways were measured. Apoptosis, migration, and cell cycle distribution were also determined. Our data revealed that the combination of TAM and FAV suppressed cell proliferation synergistically (CI < 1) and resulted in a significant change in cell migration and apoptosis. Indeed, this was associated with reduced levels of hTERT and CDK1 and shift in the cell cycle distribution. Our findings suggest that the TAM/FAV combination exhibits synergistic effects against TAMR-1 human breast cancer cells by targeting hTERT.

## Introduction

Cancer is a complex disease marked by uncontrolled cell proliferation and the capacity to expand to other tissues. It is considered the second most likely cause of death and accounted for around 10 million deaths worldwide in 2019^1^. Among all cancer types, breast cancer is the most common cancer in females, with an estimated 2.3 million new cases worldwide in 2020. Breast cancer is the fifth most prevalent cause of death due to cancer, with around 600,000 deaths worldwide in 2020^2^. Patients being treated for cancer have a four-fold increased risk of viral infection and a ten-fold increased incidence of death^3^.

Endocrine therapy is crucial in breast cancer treatment. Appropriate treatment is implemented according to the type of hormone receptor expressed on the cell surface^4^. Tamoxifen (TAM) is considered the drug of choice for estrogen receptor-positive patients. TAM works by competing with estrogen for estrogen receptor binding in the breast tissue; thus, abolishing its consequent effects on tumor tissues^5^. Moreover, TAM is an inexpensive drug, which increased its prominent role. TAM has played a central role in saving many lives in addition to increasing the survival rate of patients with breast cancer around the globe^6^. Nevertheless, this therapy is limited by the development of TAM resistance and progression to metastasis. Around 20–30% of breast cancers are resistant to TAM after 3–5 years of treatment^7^; therefore, effective strategies are required to decrease TAM resistance. TAM resistance involves a number of signaling pathways, cell cycle regulators, growth factors, autophagy, and transcription factors that control estrogen receptor expression^8–10^. Interestingly, it has been reported that the suppression of telomerase rendered cells more sensitive to anticancer drugs regardless of their mode of action^11–13^. Telomerase is a ribonucleoprotein enzyme complex that prevents telomere shortening during cell division cycles. Human telomerase reverse transcriptase (hTERT) is a crucial catalytic member of the enzyme complex that adds DNA telomeric repeats to the ends of chromosomes to prevent telomere shortening^14^. Elevated levels of telomerase have been reported in 85% of human carcinomas and 90% of breast cancers^13–15^. hTERT has been found to be overexpressed in resistant cancer cells^16,17^. Cell immortality correlates with the maintenance of telomere length and the restoration of hTERT activity. Therefore, inhibiting the catalytic activity of hTERT in breast cancer cells may induce telomere shortening and thus block tumor cell proliferation^18,19^. Indeed, the combination of anticancer drugs with telomerase inhibitors has been found to effectively kill breast cancer cells and resensitize cells to anticancer agents^13^. Several telomerase inhibitors have been studied, including immunotherapeutic agents, antisense oligonucleotides, and hTERT inhibitors^21^. Although hTERT is an RNA-dependent DNA polymerase, recent 3-D structural analyses have revealed that the hTERT protein shares structural similarity with viral RNA-dependent RNA polymerases (RdRps), including the typical right-handed architecture^21,22^. Therefore, we propose that RdRp inhibitors may elicit a potent antitumor effect.

Recently, favipiravir (FAV), an RdRp inhibitor, has gained increased attention due to its use in several countries, including Japan, Italy, Russia, Turkey, and Egypt for the treatment of COVID-19 infection with emergency approval^23,24^. In addition, FAV is effective against a variety of RNA viruses, including Ebola, norovirus, respiratory syncytial virus, rhinovirus, and influenza^26^. In 2014, Japan licensed the use of FAV (brand-name, Avigan) for the treatment of avian flu and neuraminidase inhibitor-resistant influenza^26,27^. It is worth noting that various antiviral drugs have shown promise in the prevention and treatment of cancer^29^. Repurposing of antivirals for cancer treatment is based upon the fact that cancer patients, due to their immunosuppressant state, often receive antimicrobials for prophylaxis or treatment against different infectious agents in addition to their chemotherapeutics. Interestingly, cancer patients receiving these combined treatment regimens, have shown a higher survival rate and better outcome than patients receiving chemotherapy alone^30^. Hence, combining antiviral agents with anticancer drugs for combating tumor progression is becoming more attractive due to synergistic anticancer effects, decreasing the probability of resistance development and side effects.

Furthermore, cyclin-dependent kinase 1 (CDK1), a serine/threonine kinase, is responsible for the phosphorylation of hTERT which is substantial for hTERT-mediated RdRp activity and has been reported to be associated with tumor aggressiveness^31^.

Additionally, telomerase activity is also affected by tumor necrosis factor-α (TNF-α), a major inflammatory cytokine that is significantly expressed in resistant breast cancer^32^ by causing hTERT to bind to NF-B p65 to translocate from the cytoplasm to the nucleus, a crucial step in telomere elongation induction^31,32^. TNF-α regulates the mTOR pathway as assessed by the phosphorylation status of the mTOR substrate, 4E-BP1^33,34^. mTOR mediates the phosphorylation of 4E-BP1, leading to the dissociation and release of eIF4E from 4E-BP1, enabling eIF4E to promote cap-dependent translation, resulting in increased protein synthesis of oncogenic mRNA^36^. Most cancer cells, including breast cancer cells, have increased eukaryotic translation initiation factor 4E (eIF4E) expression, which leads to the upregulation of cancer-promoting genes^37^ and overexpression of eukaryotic initiation factor 4E-binding protein (4E-BP1), which is linked to endocrine resistance and poor prognoses^29^.

The role of some antivirals such as zidovudine, abacavir and lamivudine in sensitization of cancer cell lines to radiation was previously reported^38^, yet the effect of FAV on cancer cells and its mechanism of action was not yet investigated. Therefore, the present study focuses on investigating for the first time the proposed role of FAV alone and its combination with TAM in inducing breast cancer cell death in TAM-resistant MCF-7 breast cancer cell lines (TAMR-1). The effect of TAM or FAV, and the combination of both drugs on the migratory potential, proliferative capacity, cell cycle, and apoptotic activities of MCF-7 and TAMR-1 cells was investigated. The levels of hTERT, in addition to hTERT-targeted pathways regulators; CDK-1, 4E-BP1, eIF4E, and TNF-α were measured in resistant and sensitive MCF-7 cells.

## Results

### Tamoxifen and favipiravir monotherapy decreased the viability of MCF-7 and TAMR-1 cancer cells while cotreatment potentiated the effects

**Figure 1:**
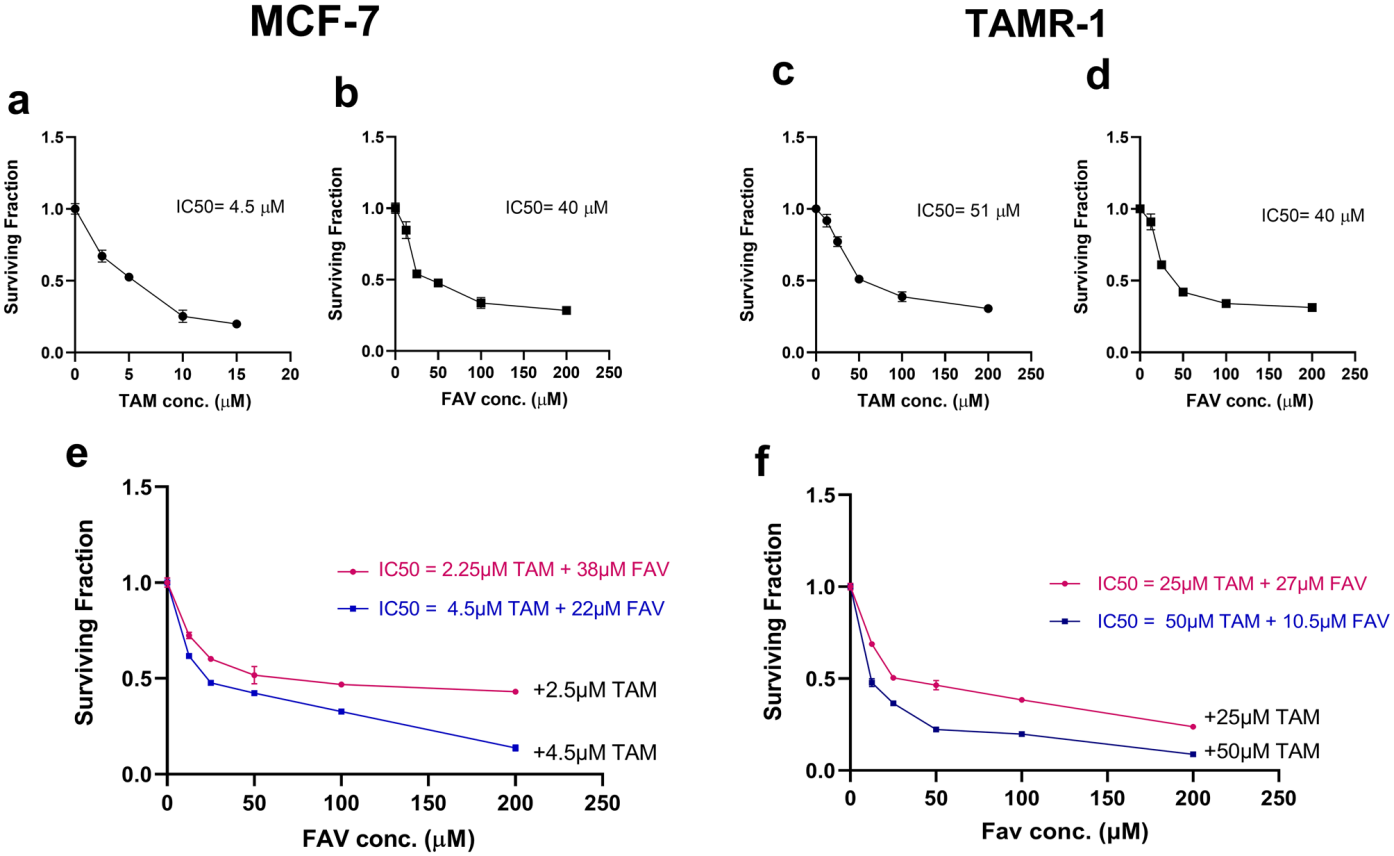
The surviving fractions of MCF-7 and TAMR-1 cells with different concentrations of TAM (**a**,**b**) or FAV (**c**,**d**), or their combination (**e**,**f**), respectively, for 48 h. The values shown are the mean ± SD of two independent experiments performed in triplicate. The surviving fraction is the percentage of viable cells in comparison to the control. Surviving fraction = optical density (treated cells)/optical density (untreated cells).

As a first step, the viability of the carcinoma cell lines in the presence of TAM or FAV or their combination was determined using sulforhodamine B (SRB) assay. It was observed that FAV and TAM had an inhibitory effect on MCF-7 and TAMR-1 breast cancer cells in a dose-dependent manner following 48 h of drug incubation. The average IC50 values of TAM and FAV in MCF-7 cells were 4.5 μM and 40 μM, respectively (Fig. 1a,b), whereas the average IC50 values of TAM and FAV in TAMR-1 cells were 51 μM and 40 μM, respectively (Fig. 1c,d). Although MCF-7 cells are more sensitive to TAM monotherapy than TAMR-1 cells, both cell lines showed the same sensitivity toward FAV with equal IC50 values of 40 μM.

In both cell lines, there was a significant decrease in viability in all combination-treated samples as compared to monotherapy (Fig. 1e,f). When TAM (50 μM) and FAV (50 μM) were combined to treat TAMR-1 cells, the inhibitory rate reached 78%, whereas treatment with TAM alone achieved an inhibitory rate of 48%. In MCF-7 cells, treatment with TAM (2.25 μM) in combination with FAV (100 μM) inhibited cell growth by approximately 16% when compared to TAM alone.

CompuSyn software was used to analyze the effect of the combination’s interaction to see whether combinations would have a synergistic effect and result in a greater reduction in cell growth than the single drugs. For both drugs, synergism equivalent to a combination index (CI) of < 1 invariably resulted in a favorable dose-reduction index of > 1 (see Supplementary Fig.*** S1 online). As a result, for the subsequent studies using TAMR-1 cells, a 50 μM FAV and 50 μM TAM combination regimen was chosen since it had the lowest CI (0.75) when compared to other combinations.

### The effect of TAM (50 μM), FAV (50 μM), or their combination on the gene expression of hTERT and CDK1 in TAMR-1 cells

**Figure 2:**
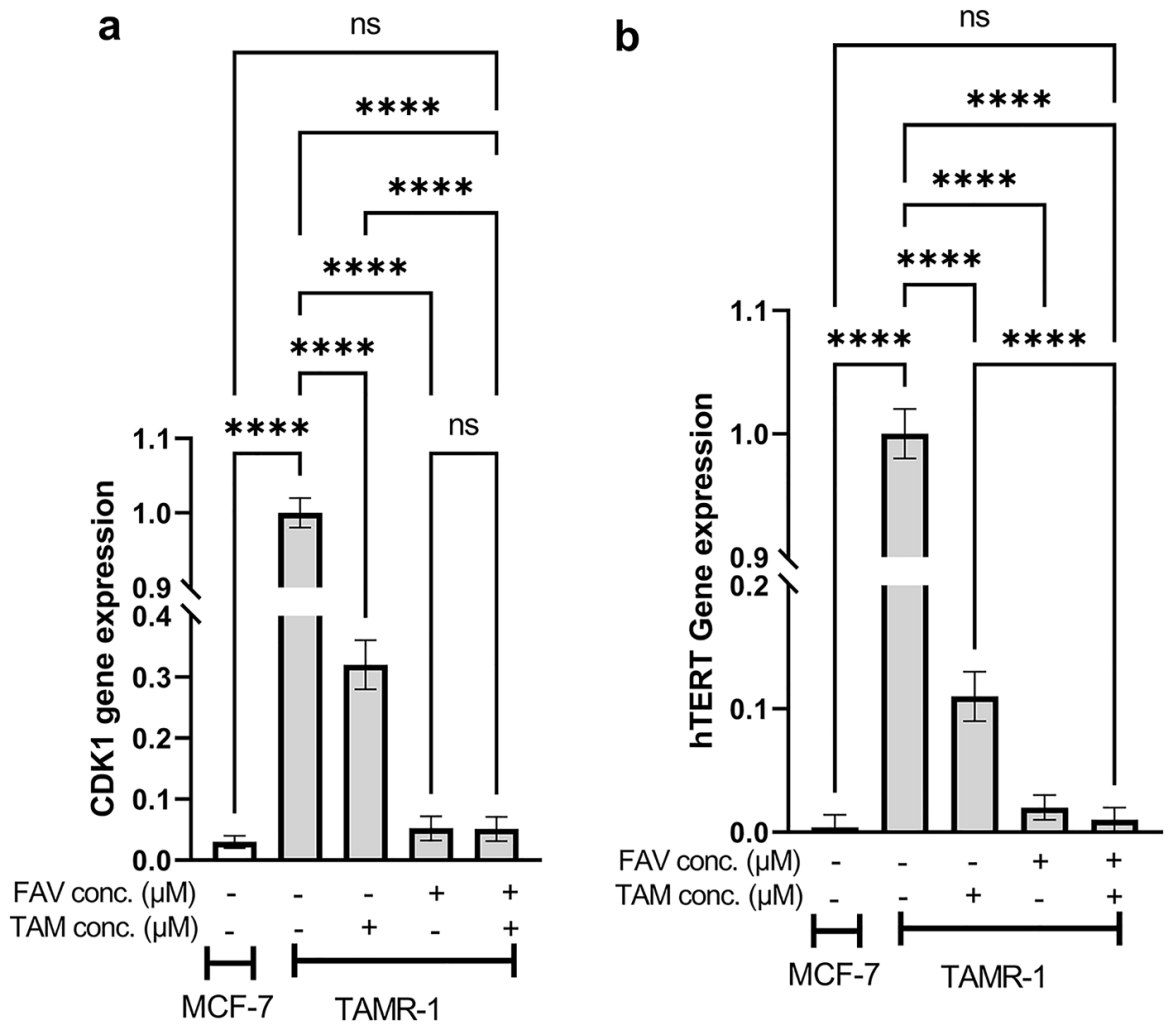
qPCR analysis for the expression levels of CDK1 (**a**) and hTERT (**b**) in MCF-7 and TAMR-1 cells. Data represented in the bar graphs are from three independent experiments. The statistical differences were investigated using a one-way ANOVA test followed by a Games Howell posthoc test. ***Significant at *P* < 0.0001. ****Significant at *P* < 0.00001.

To investigate the putative suppressive role of FAV on hTERT expression and consequent synergistic effect in TAMR-1 cells, the gene expression levels of hTERT and CDK1 were assessed after treating cells with TAM or FAV or their combination. The gene expression levels of hTERT and CDK1 were found to be significantly higher in TAMR-1 cells than in MCF-7 cells (*P* < 0.0001) (Fig. 2), indicating that the upregulation of these proteins correlates with TAM resistance. Each drug and their combination significantly decreased hTERT and CDK1 expression when compared to the TAMR-1 control (*P* < 0.0001). Moreover, the combination significantly decreased the hTERT and CDK1 gene expression levels when compared to TAM monotherapy.

### The effect of TAM (50 μM), FAV (50 μM), or their combination on TNF-α, elF4E, and 4E-BP1 levels in TAMR-1 cells

**Figure 3:**
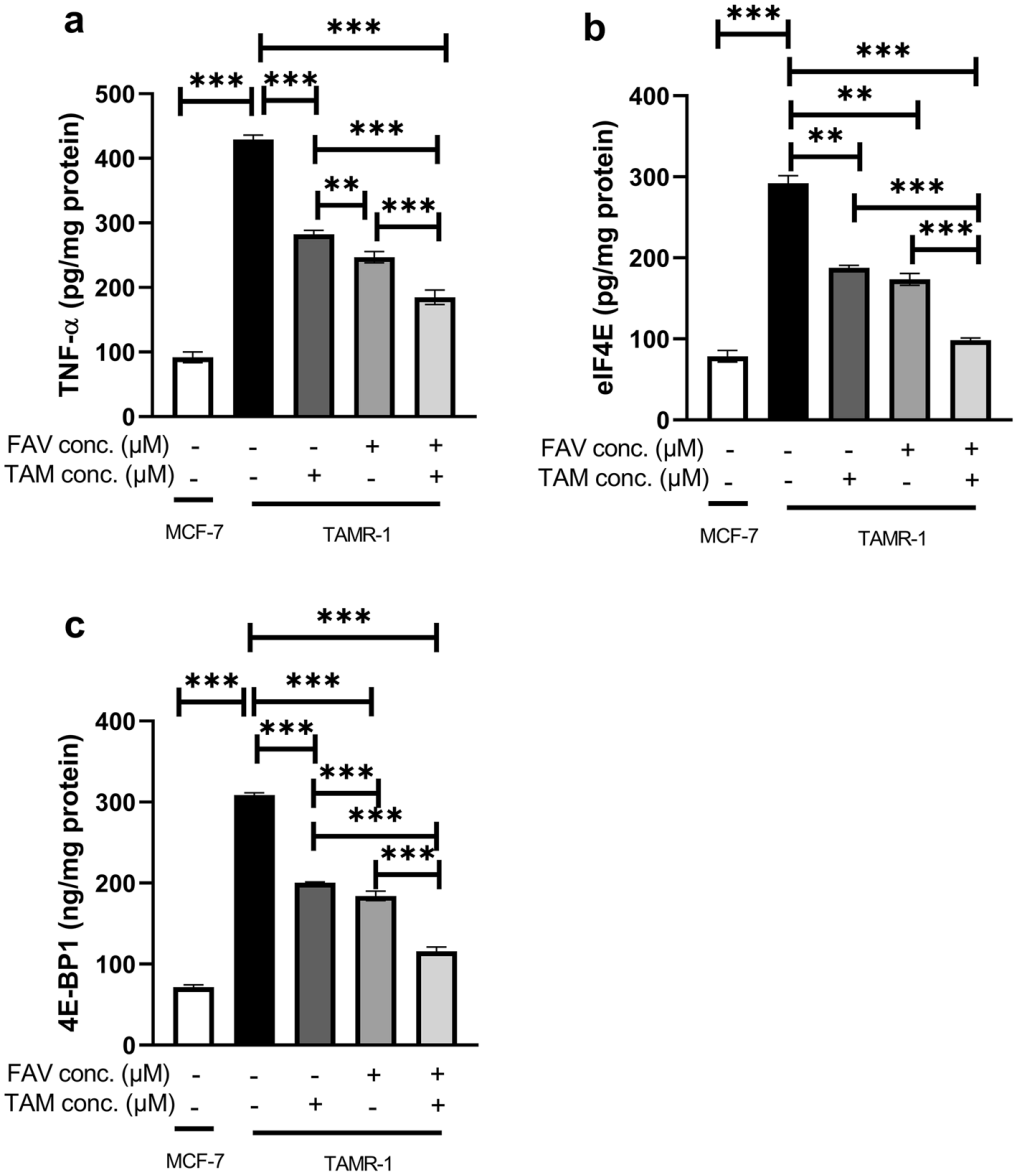
TNF-α (**a**), eIF4E (**b**), and 4E-BP1 (**c**) levels in MCF-7 and TAMR-1 cells. Data represented in the bar graphs are from three independent experiments. One-way ANOVA test followed by Games Howell post hoc test was used to examine statistical differences. **Significant at *P* < 0.001. ***Significant at *P* < 0.0001.

Figure 3 shows that TAMR-1 cells expressed significantly higher levels of TNF-α, eIF4E, and 4E-BP1 compared to MCF-7 cells (*P* < 0.0001). In TAMR-1 cells, the levels of TNF-α, eIF4E, and 4E-BP1 significantly decreased after TAM/FAV monotherapy and the combined therapy compared to the untreated TAMR-1 cells. In addition, FAV treatment induced lower levels of TNF-α, eIF4E, and 4E-BP1 expression when compared to TAM. The combination significantly lowered the levels of TNF-α, eIF4E, and 4E-BP1 when compared to treatment with each drug alone (*P* < 0.0001). Interestingly, their levels in the combination-treated TAMR-1 cells did not differ when compared to the untreated MCF-7 cells.

### The effect of TAM (50 μM), FAV (50 μM), or their combination on wound healing in TAMR-1 cells

**Figure 4:**
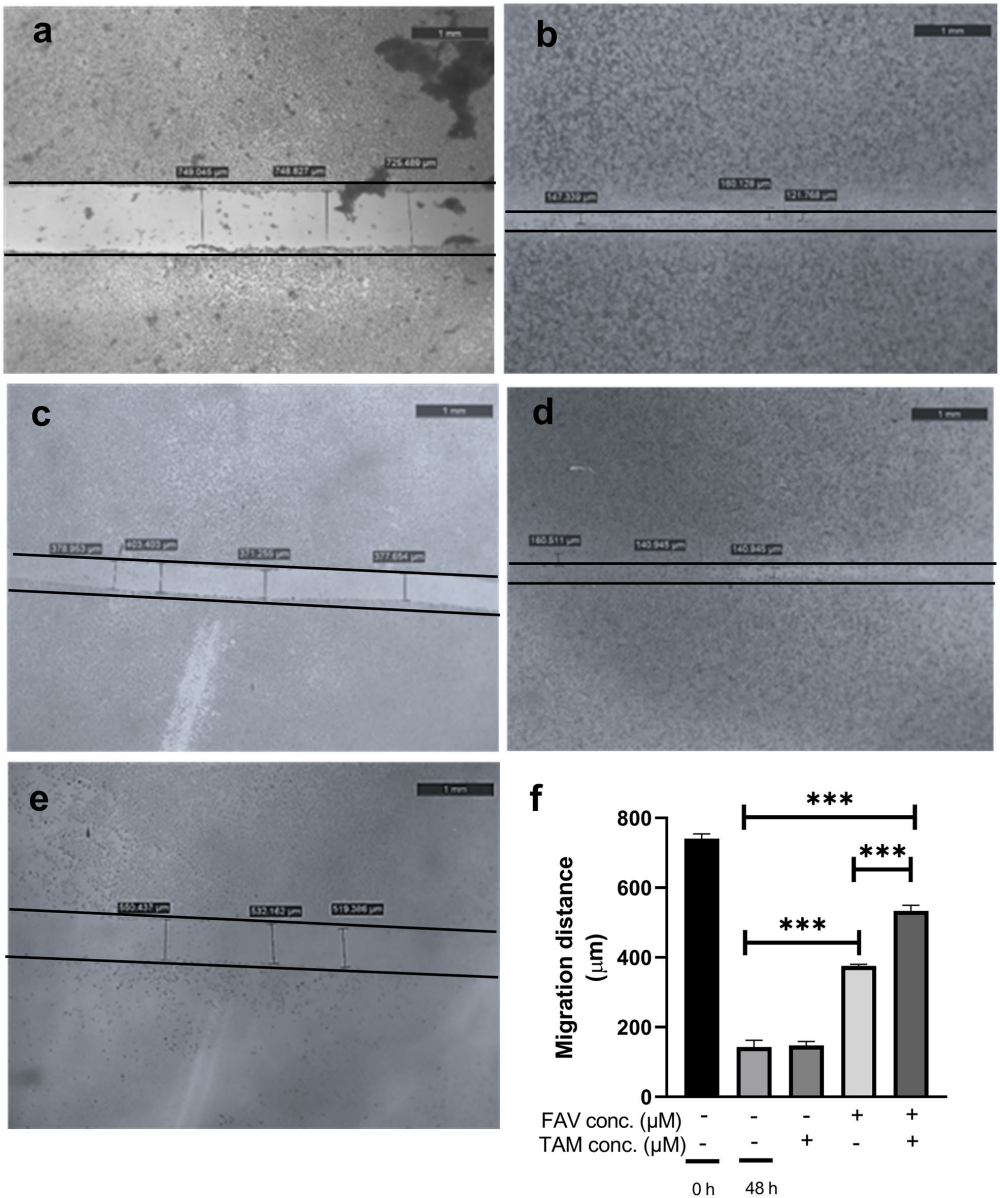
Wound healing assay of the treated TAMR-1 cells. Panel (**a**) represents the control at 0 h, (**b**) represents the control at 48 h. (**c**–**e**) represent TAM, FAV, and their combination at 48 h, respectively, at concentrations of 50 μM each. Panel (**f**) shows the bar graph quantifying the migration of the cells for each drug. Plotted points are the mean representation of the data ± SD. The statistical differences were investigated using a one-way ANOVA test followed by a Games Howell post hoc test. ***Significant at *P* < 0.0001.

The ability of TAMR-1 cells to migrate was examined using a wound healing test to see if FAV may improve TAM treatment and prevent TAMR-1 cell migration. Figure 4a illustrates TAMR-1 control cells at 0 h. When compared to the control at 48 h (Fig. 4b), TAM monotherapy significantly reduced migratory capability (Fig. 4c), while FAV monotherapy (Fig. 4d) had no significant effect on the wound healing ability of TAMR-1 cells (*P* > 0.05). Interestingly, the combination of FAV (50 μM) and TAM (50 μM) resulted in a substantial delay in wound healing when compared to FAV and TAM monotherapies (*P* < 0.0001) (Fig. 4e).

### The effect of TAM (50 μM), FAV (50 μM), or their combination on apoptosis induction in TAMR-1 cells

**Figure 5:**
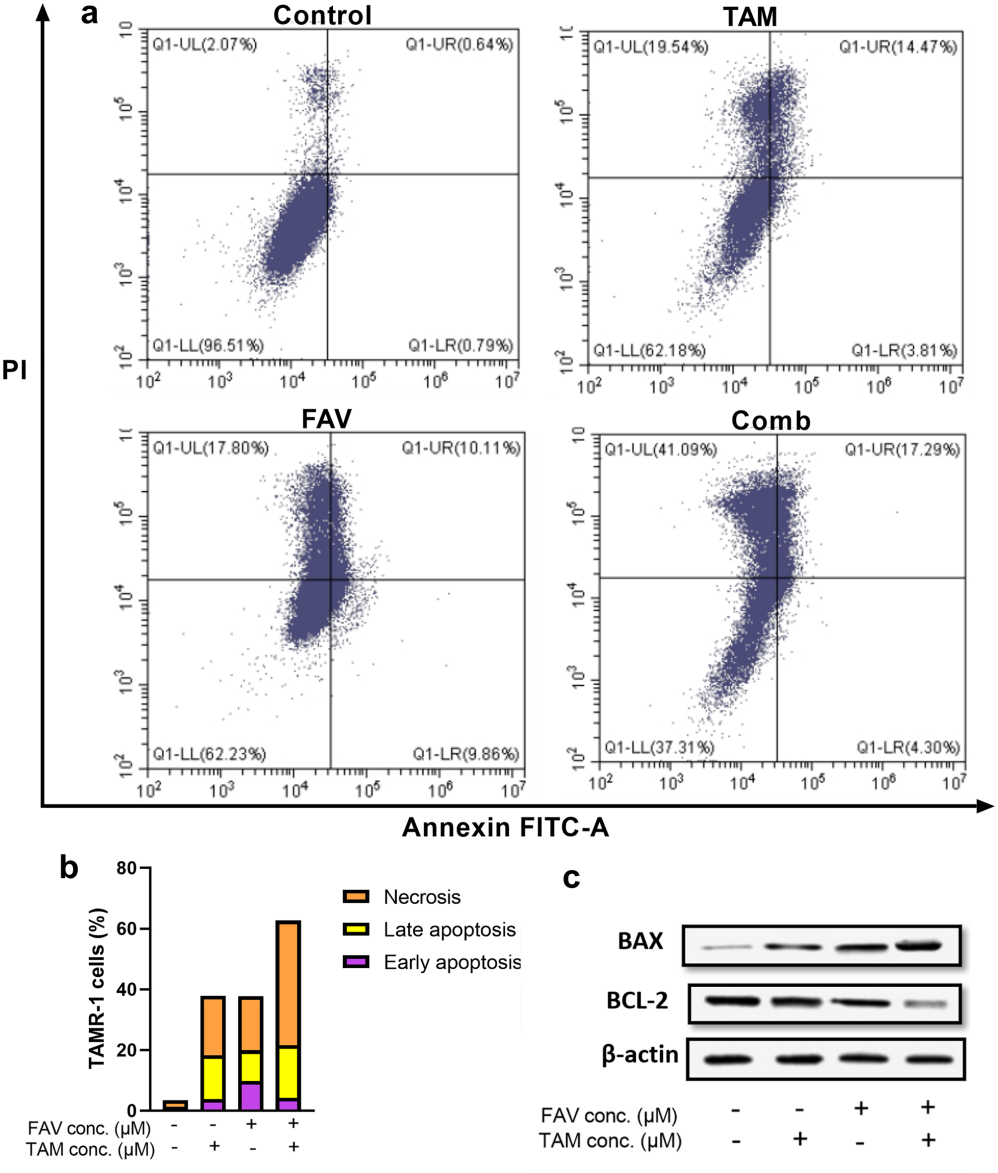
The effect of TAM or FAV or their combination on TAMR-1 cell apoptosis. (**a**) TAMR-1 early apoptotic cells, late apoptotic cells, and necrotic cells were detected by flow cytometry at 48 h using annexin V/propidium iodide (PI). (**b**) The bar chart shows a higher proportion of late apoptotic and necrotic cells after combination therapy compared to monotherapy with either drug. Cell numbers (percentages) from four different quadrants are represented in the representative dot plots (UL; necrosis, UR; late apoptosis, LL; live, LR; early apoptosis). (**c**) The expression levels of BAX and BCL-2 in TAMR-1 cells using Western blot analysis. Results are expressed as mean ± standard deviation for three independent experiments. **Significant at *P* < 0.001. ***Significant at *P* < 0.0001. The original blots are presented in Supplementary Fig. S2.

To investigate whether TAMR-1 cells undergo apoptosis upon treatment with tamoxifen, favipiravir, or their combination, cell staining with annexin V and propidium iodide (PI) was performed and cells were analyzed using flow cytometry. Induction of cell apoptosis of TAMR-1 cells treated with TAM (50 μM) or FAV (50 μM) monotherapy or their combination was observed at 48 h (Fig. 5). In comparison to the single-drug treatment groups, the number of late apoptotic and necrotic cells in the combination-treatment group was increased. The percentage of TAMR-1 cells that were found to be necrotic or apoptotic is shown in Fig. 5b. To confirm that combination therapy induces apoptosis in TAMR-1 cells, the levels of the proapoptotic protein, BAX, and the antiapoptotic protein, BCL-2, were measured (Fig. 5c). The Western blot results revealed that the expression level of BAX was significantly increased, while the expression of BCl-2 was decreased after treatment with the drug combination (*P* < 0.05). These outcomes matched the outcomes of the SRB test, suggesting that the addition of FAV to TAM has a synergistic effect on TAMR-1 cells.

### The effect of TAM (50 μM), FAV (50 μM), or their combination on cell cycle distribution of TAMR-1 cells

**Figure 6:**
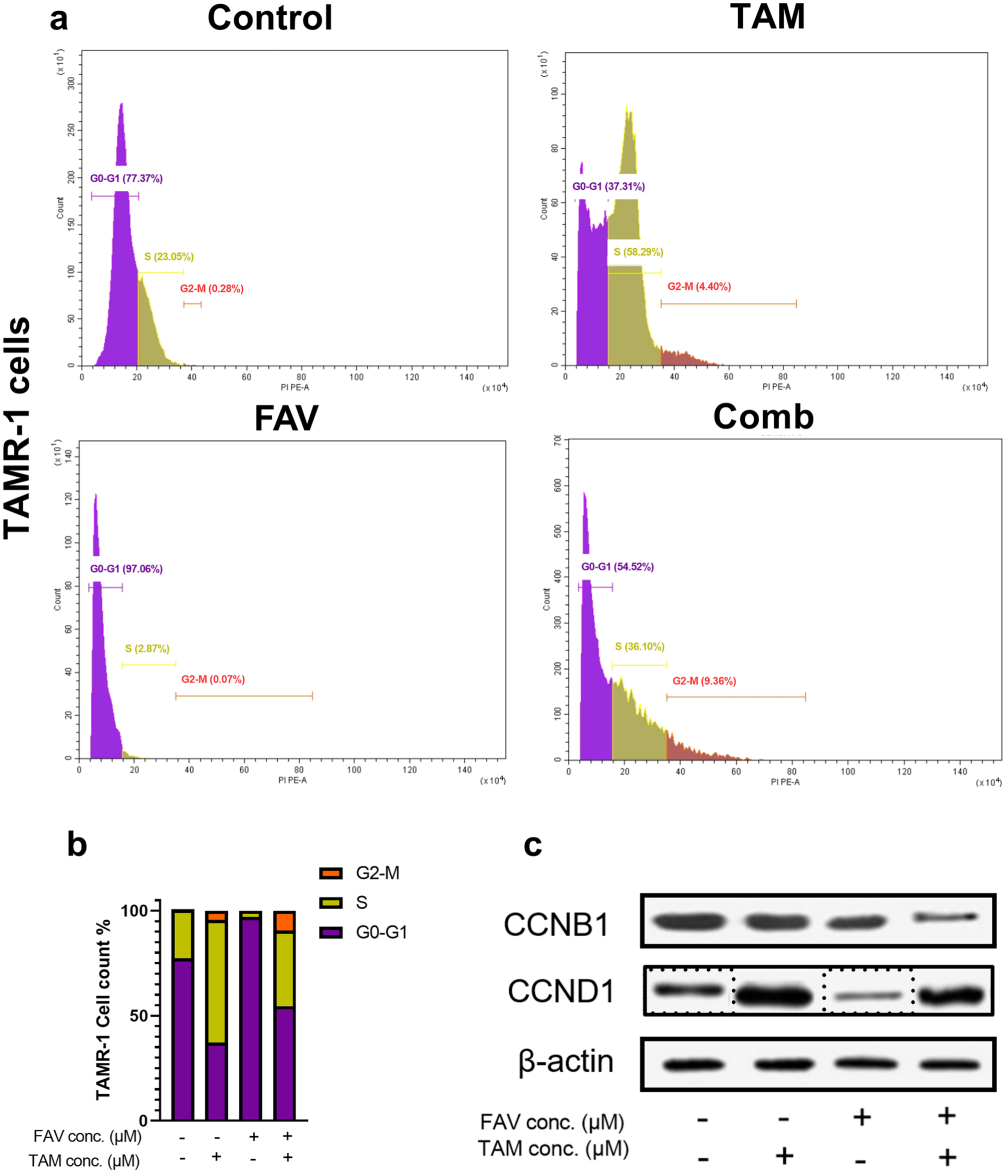
Flow cytometry and Western blot analysis of the effect of tamoxifen, favipiravir, or their combination on cell cycle regulation in TAMR-1 cells. (**b**) Flow cytometry results of PI-stained TAMR-1 cells showing the percentage of the cells in G0/G1 (purple), S (green), and G2/M (orange) phases (**b**) bar graphs representing cell cycle distribution percentages between the different drugs. (**c**) Expression levels of CCNB1 and CCND1 in TAMR-1 cells using Western blot analysis. Results are expressed as mean ± standard deviation for three independent experiments. **Significant at *P* < 0.001. ***Significant at *P* < 0.0001. The original blots are presented in Supplementary Fig. S3.

The cell cycle distribution was assessed by flow cytometry after 48 h to further determine if the antiproliferative impact caused by TAM, FAV, or their combination is linked to cell cycle arrest. Figure 6 shows that there was a clear alteration in the distribution of different phases when cells were treated with TAM, FAV, or their combination. Remarkably, TAM arrested the cell cycle at the S (58.29%) and G_2_/M (4.40%) phases, while FAV arrested the cell cycle at the G_0_/G_1_ phase (97.06%). The combination of TAM and FAV significantly increased the percentage of cells accumulated at the G_2_/M phase (9.36%) compared to the control (0.28%) and each drug alone (TAM, 4.4%; FAV, 0.07%). When compared to the control group, TAM-treated cells showed a significant increase in cyclin D1 (CCND1) levels, while FAV treatment significantly decreased the CCND1 level. Interestingly, combining the drugs significantly increased the CCND1 level compared to the control but CCND1 showed a significant decrease compared to its corresponding level in TAM-treated cells. Moreover, the combination significantly decreased cyclin B1 (CCNB1) levels compared to the control and to each drug alone.

## Discussion

Breast cancer is the most frequent cancer in females and the main cause of death from cancer^2^. TAM is one of the most chemotherapeutic medications used to treat breast cancer, however with time, cancer cells become resistant to TAM^8–11^. Some genes have been found to be associated with mechanisms of tamoxifen resistance^39^. Thus, exploration of more effective strategies is demanded to overcome resistance in breast cancer cells, improve TAM efficacy, and decrease undesirable side effects^9,38–40^. Diverse attempts are being investigated to prevail this impediment including novel therapeutic agents, immunotherapy, gene therapy, and combination therapy^10,40,41,45^. Nonetheless, clinical outcomes are still mediocre urging a thorough understanding of the underlying chemoresistance mechanisms, and improvement of treatment approaches.

A known resistance mechanism focuses on hTERT overexpression^16,17^. hTERT promotes cancer cell proliferation, angiogenesis, migration, and metastasis^39,40^. Elevated hTERT levels have been related to poor prognoses in solid tumors, including gastric, cervical, lung and breast cancer^48^. hTERT has previously been shown to have RdRp activity, which is required for tumor development^42,43^.

FAV, an RdRp inhibitor, is used against various RNA virus infections. In this study, FAV was combined with TAM due to its RdRp inhibitory effects and the structural similarity between hTERT and RdRp^44,45^. In addition, infection is more common in patients with cancer than in the normal population^46,47^. It has been reported that knockdown of hTERT inhibits cancer proliferation and attenuates resistance to radio- and chemotherapy^16,48^. Moreover, the phosphorylation of hTERT by CDK1 participates in cancer development^31^. CDK1 is crucial in cell cycle control and is considered as a key element of mitosis^56^. CDK1 inhibitors is an important target for cancer treatment and are already involved in some clinical trials^51,52^. Also inhibiting CDK1 has been reported to induce apoptosis selectively in MYC-dependent breast cancer cells^59^. According to our gene expression analysis, TAMR-1 cells had higher levels of hTERT and CDK1 than MCF-7 cells. Meanwhile, treatment with TAM, FAV, and their combination decreased the expression levels of hTERT and CDK1 in TAMR-1 cells significantly when compared to the TAMR-1 control. In addition, compared to the single agents, the medication combination significantly reduced the expression of CDK1 and hTERT. Interestingly, the combination restored hTERT and CDK-1 levels in TAMR-1 cells to those in MCF-7 cells. These findings are in agreement with a prior report stating that the downregulation of hTERT in MCF-7 cells rendered cells more sensitive to chemotherapeutic drugs^17^. Combining metformin and silibinin inhibited T47D breast cancer cells’ proliferation synergistically via inhibition of hTERT^60^. Moreover, a combination therapy of roscovitine, a pan-CDK-1 inhibitor, followed by doxorubicin enhanced doxorubicin efficiency and reduced toxicity in triple-negative breast cancer^61^.

The possible FAV-induced mechanisms underlying the downregulation of hTERT expression and synergistic potentiation of breast cancer cell death was also investigated in this study. hTERT was found to be regulated by wingless (Wnt), mammalian target of the rapamycin (mTOR), and mitogen-activated protein kinase (MAPK) signalling pathways^54,55^. TNF-α controls protein synthesis by activating MAPK and regulates telomerase activity by translocating hTERT protein coupled to NF-B p65 from the cytoplasm to the nucleus, a substantial step for inducing telomere elongation^33^. MAPK then causes the eukaryotic initiation factor, eIF4E, to be phosphorylated. Moreover, TNF-α regulates the mTOR pathway as assessed by the phosphorylation status of the mTOR substrate, 4E-BP1^33,34^. mTOR mediates the phosphorylation of 4E-BP1, leading to the dissociation and release of eIF4E from 4E-BP1, enabling eIF4E to promote cap-dependent translation, resulting in increased protein synthesis of oncogenic mRNA^36^. Dependent on the tumor microenvironment, 4E-BP1 plays a regulatory role by selectively modulating the translation of particular transcripts that function as drivers of cancer cell proliferation and progression by adapting the tumor to metabolic and genotoxic stress^64^.

Moreover, Zhang et al.^65^ reported that high levels of TNF-α promote chemoresistance in breast cancer cells. 4E-BP1 has also been linked with poor prognoses and drug resistance in patients with cancer. Knockdown of 4E-BP1 in breast cancer cells has been found to result in substantial decreases in cell proliferation^66^. eIF4E is a known protumorigenic factor, with elevated expression or activity in a variety of malignancies. High expression of eIF4E indicates poorer recurrence-free survival levels^67^. Moreover, CDK1 was recently reported to phosphorylate 4E-BPI in the absence (or low levels) of mTOR kinase as an alternative method to maintain phosphorylation during mitosis^68^. Our data revealed that TAMR-1 cells showed significantly higher levels of TNF-α, eIF4E, and 4E-BP1 in comparison to MCF-7 cells. Moreover, the combination of TAM and FAV significantly lowered the levels of TNF-α, eIF4E, and 4E-BP1 when compared to each drug alone.

Our study demonstrates that a combination of TAM and FAV can synergistically inhibit cell proliferation and increase apoptosis in MCF-7 and TAMR-1 cells. TAM-treated TAMR-1 cells showed a higher IC50 than TAM-treated MCF-7 cells, whereas FAV showed a consistent IC50 across both cell lines. These results are in agreement with prior reports that demonstrated that TAM treatment inhibited cell growth and apoptosis in MCF-7 cells, and that breast tumors developed resistance to TAM^8,60^. Furthermore, it has been reported that FAV has an inhibitory effect on A549 lung cancer cells^72^. Our results showed that the percentage of apoptotic and necrotic cells in the combination-treated group were increased compared with the single-drug treatment group. TAM has been reported to induce apoptosis in MCF-7 cells^3^. Moreover, downregulating hTERT results in the induction of apoptosis and the suppression of cell viability in MCF-7 cells^73^. Two apoptotic pathways exist: the extrinsic and intrinsic pathways. The intrinsic pathway, also known as the mitochondrial pathway, is controlled by BCL-2 family proteins, including the proapoptotic protein, BAX, and the antiapoptotic protein, BCL-2. In cancer cells, BCL-2 is usually upregulated, inhibiting the proapoptotic BAX; therefore, inhibiting apoptosis^63,64^. Our data demonstrate that using a combined therapy of TAM and FAV promotes downregulation of BCL-2 antiapoptotic proteins and the upregulation of BAX proapoptotic proteins in TAMR-1 cells. The increased expression of BAX protein, associated with BCL-2 decrease, in cells incubated with combined doses of FAV and TAM illustrates that combination therapy ameliorates the BAX effect, leading to the induction of the intrinsic apoptosis pathway. Similarly, a previous study showed that combining TAM with histone deacetylase inhibitors in TAMR-1 cells induced apoptosis by downregulating BCL-2, suggesting that a high level of Bcl-2 is a key driver of TAM resistance^62^.

The distribution of the TAMR-1 cells in the cell cycle was determined for a better understanding of the mechanisms of TAM and FAV underlying cell growth inhibition. The analysis of flow cytometry data revealed that TAM and its combination with FAV caused cells to assemble at the S and G_2_/M phases, while simultaneously depleting G_0_–G_1_-phase cells. Conversely, FAV triggered cells to aggregate at the G_0_–G_1_ phase. The drugs inhibited the growth of MCF-7 breast cancer cells by delaying the cell cycle transition. However, different studies have found that TAM causes MCF-7 cells' cell cycle to stop at the G0–G1 stage^65,66^. Western blot analysis of CCND1 and CCNB1 showed that the drug combination led to a significant increase in CCND1 when compared to the control group, while its level was lower than in TAM-treated cells. It was previously reported that CCND1 overexpression is associated with better outcomes for patients with breast cancer but its overexpression is linked to TAM resistance^78,79^. CCND1 phosphorylates and inactivates retinoblastoma protein, allowing cells to progress from G1 phase to S phase^79^. High CCNB1 cyclin B1 expression is associated with poor survival in breast cancer^80^. This agrees with our results, which showed that the combination significantly decreased its levels.

Our data demonstrated a significant reduction in cell migration of TAMR-1 cells when combination treatment of TAM and FAV was implemented compared to each drug alone. This result was in accordance with a previous study that showed a slowdown in wound healing by TAM in MCF-7 cells in a dose-dependent manner^81^. Moreover, it was previously reported that hTERT increases cell migration and its regulation with miR-138-5p suppresses cell growth and migration^82–84^.

**Figure 7:**
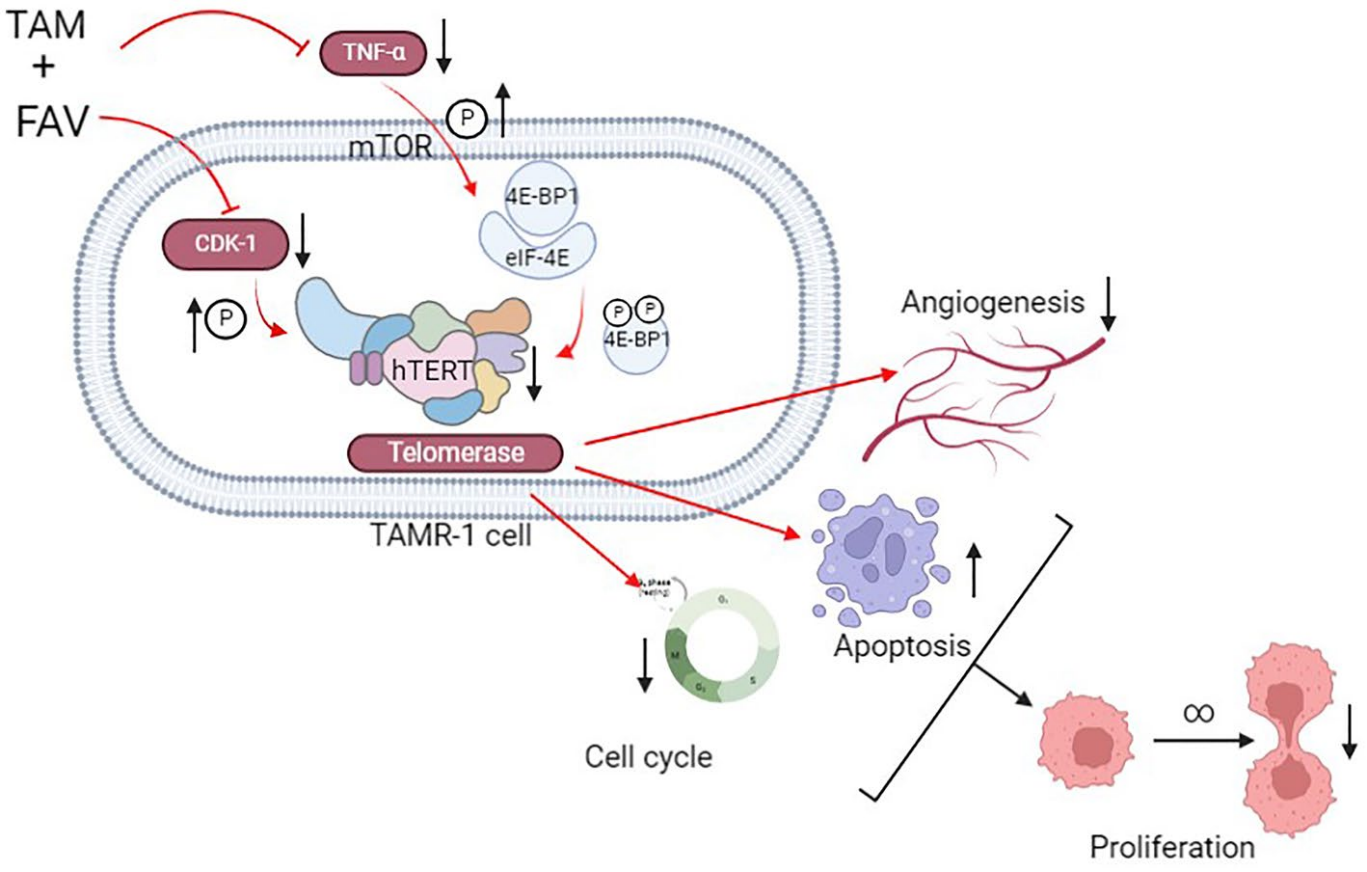
Graphical abstract displaying the role of tamoxifen and Favipiravir combination on TAMR-1 breast cancer cells’ proliferation and angiogenesis.

In conclusion, hTERT overexpression is a possible mechanism contributing to TAM therapy resistance. Our findings confirmed that FAV synergistically enhances the anticancer effect of TAM in TAMR-1 cells by downregulating hTERT, TNF-α, eIF4E, and 4E-BP1 expression. The combination of TAM and FAV elicits inhibition of proliferation and invasiveness as well as inducing apoptosis and a shift in the cell cycle distribution (Fig. 7). Thus, our results identified a new mechanism of action for FAV in TAM-resistant breast cancer cells, suggesting that combining FAV with TAM might be an effective therapeutic option to overcome endocrine resistance in breast cancer. Moreover, our data suggests that hTERT could be a potential therapeutic target for the treatment of TAM resistance in breast cancer.

## Materials and methods

### Drugs and chemicals

TAM was purchased from Amriya Pharm. Ind. (Alexandria, Egypt), and FAV was acquired from Liptis Pharmaceuticals (Cairo, Egypt). Both drugs were stored at room temperature and before use they were dissolved in RPMI-1640 medium.

### Cancer cell lines

The American Type Culture Collection provided the MCF-7 human breast cancer cell line (Minnesota, USA.). The National Cancer Institute in Cairo, Egypt, used serial subculturing to keep cell lines alive. The human tumor cell lines have been grown and maintained in RPMI-1640 media supplemented with 10% fetal bovine serum (FBS) and 1% penicillin/streptomycin. The TAMR-1 breast cancer cell line was generated by subjecting the MCF-7 cell line to mounting concentrations of TAM over a one year period to induce resistance.

### Cell culture and drug assays

In 75-cm^2^ flasks, cells were cultured in RPMI-1640 medium enriched with 10% FBS and antibiotics. Cells were subcultured to 70% confluence at 37 °C and 5% CO_2_. After aspirating the media, cells were incubated with fresh medium containing diverse concentrations of TAM or FAV or a mixture of the two, for 48 h at 37 °C and 5% CO_2_. Supernatants and cells were collected for different concentrations and the control. Cells were harvested with 0.25% trypsin–EDTA, lysed with lysis buffer, and kept at − 80 °C.

### Sulforhodamine-B (SRB) (cytotoxicity) assay

MCF-7 and TAMR-1 cells were seeded at a concentration of 2 × 10^3^ cells/well in 96-well plates with RPMI-1640 media, 10% FBS, and antibiotics. Plates were maintained at 37 °C in a 5% CO_2_ atmosphere overnight. Cells were then treated with different concentrations of TAM or FAV (0–200 μM) and incubated for 48 h. IC50 values were generated using GraphPad Prism 8.4.2. The combination regimen was designed using the IC50 and half IC50 of TAM and increasing doses of FAV (0–200 μM). The SRB assay was used to perform the cytotoxicity test. The optical density for each well was determined using ELISA microplate reader (Sunrise, Tecan, Germany). Control cells were incubated without the drug. Using CompuSyn software, we employed the CI approach^85^ to examine if the cytotoxic interactions of TAM and FAV were synergistic, additive, or antagonistic.

### hTERT and CDK-1 gene expression

Total RNA was extracted from treatment and control samples using a total RNA purification kit (Jena Bioscience, Munich, Germany). A cDNA archive kit (Applied Biosystems, Foster City, California, USA) was used to transform RNA to complementary DNA. GoTaq PCR master mix (Promega Co., Madison, USA) was used for qPCR, which included 25 µL of master mix, 1 µL of forward and reverse primers, 1 µL of cDNA, and 0.25 µL of CXR reference dye then completed to a final volume of 50 µL. All analyses were conducted in triplicates on a 7500 qPCR system (Applied Biosystems, Foster City, CA, USA). The 2^−ΔΔCt^ method was employed for data analysis. Outcome results were recorded as relative expression levels after being adjusted to GAPDH^86^. The primer sequences used were recorded in Table 1.

**Table 1:** Primer sequences.

Gene	Forward primer	Amplicon size (bp)	Accession #
hTERT	F-5′-GCAAGTTGCAAAGCATTGGA-3′ R-5′-ACCTCTGCTTCCGACAGCTC-3′	73	NM_001193376.3
CDK-1	F-5′-AAGCTGGCTCTT GGAAATTGA-3′ R-5′-ATGGCTACCACTTGACCTGTAGTT-3′	200	NM_033379.5
GAPDH	F-5′-ACCCACTCCTCCACCTTTGA-3′ R-5′-CTGTTGCTGTAGCCAAATTCGT-3′	101	NM_001357943.2

### TNF-α, elF4E, and 4E-BP1 levels

The TNF-α level was measured in cell culture supernatants, while the 4E-BP1 and eIF4E protein levels were measured in the cell lysate. The TNF-α, 4E-BP1, and eIF4E levels in MCF-7 and TAMR-1 cells were estimated using human TNF-α ELISA kits (ab285312, Abcam, UK), human eIF4eBP1 ELISA kits (ab289651, Abcam), and human eIF4E ELISA kits (ab214564, Abcam), respectively. An ELISA reader (TP-Thermoplate Reader) was used to measure each parameter three times, following the manufacturer’s directions, at 450 nm. Total protein was measured, and the results were expressed as mean concentration per 1 mg of total protein ± standard deviation.

### Cell migration

TAMR-1 cells were seeded onto a 6-well plate. Using a sterile pipette, a single scratch was made creating a cell-free area, then the plate was rinsed to remove excess cells. Afterwards, the medium in the wells was aspirated and replaced with medium containing TAM or FAV or their combination. Controls comprised untreated wells at 0 and 48 h. An inverted microscope (DFC290, Leica, Wetzlar, Germany) was used to capture the images to analyze the size of the formed gap for each well.

### Annexin V-FITC/PI for detection of apoptosis by flow cytometry

Flow cytometry was performed to examine apoptosis and necrosis of human cancer cells. A FITC Annexin V kit was used to further investigate apoptosis. Before being washed with cold PBS, all aspirates were centrifuged for 5 min at 13,000 rpm at 4 °C. The cells were extracted and centrifuged for 5 min at 2500 rpm at 10 °C. The supernatant was discarded, and the pellet was rinsed with 1 mL PBS before centrifugation at 2500 rpm for 5 min at 10 °C. Pellets were incubated for 30 min in PBS (50 μL) with Annexin V-FITC and PI. After incubation, flow cytometry analysis was carried out in 300 μL of PBS containing incubated cells in a flow cytometer (BD Biosciences LSR II FACS, New Jersey, USA). Apoptotic cells release phosphatidylserine extracellularly, which is detectable by using annexin V-labeled fluorescence. TAM or FAV were added to TAMR-1 cells alone and in combination, with PI antibodies and annexin V. Early apoptotic cells express annexin + PI−, late apoptotic cells expressed annexin + PI+ and necrotic cells expressed annexin–PI+.

### Cell cycle analysis

Flow cytometry was utilized to assess the cell cycle in TAMR-1 cells, which were treated with FAV or TAM or their combination. After treatment, cells were trypsinized and fixed using 70% (v/v) ethanol and left overnight at 4 °C. After being rinsed twice with PBS, fixed cells were resuspended in PBS (Beyotime, Jiangsu, China) containing 50 g/mL PI and 0.1 mg/mL RNaseA. Following 30 min of incubation at 37 °C in the dark, the cells were analyzed using a flow cytometer (BD Biosciences LSR II FACS, New Jersey, USA). The cell count was estimated in each stage of the cell cycle (G0/G1, S, and G2/M).

### Western blot assay

Ice-cold lysis buffer (1% NP-40, 0.1% SDS, 0.1% sodium deoxycholate, 150 mM NaCl, 1 mM EDTA, 10 mM Tris–HCl; pH 7.4) was added to the cells to obtain the cell lysate required. To separate the protein from the cell lysate, the proteins were subjected to electrophoresis on 12% SDS–polyacrylamide gels and electrophoretically blotted to Amersham Hybond P Western blotting membranes (GE10600021 Sigma, Sigma-Aldrich, MO, USA). After blocking with 5% skim milk, the membranes were probed with primary antibodies mouse anti-β-actin and CCND1 or CCNB1 or BAX or BCL-2 monoclonal antibodies (Santa Cruz Biotechnology, Santa Cruz, California, USA) for 1 h followed by HRP-conjugated rabbit antimouse IgG or HRP-conjugated goat antirabbit IgG. Quantification of the protein-bound bands was performed by image analysis using the ChemiDoc MP imaging system (v3, Hercules, California, USA).

### Supplementary Information

Supplementary Figures.

## Data Availability

The datasets generated during and/or analyzed during the current study are available from the corresponding author.
