# Supplementary material for: Favipiravir, an antiviral drug, in combination with tamoxifen exerts synergistic effect in tamoxifen-resistant breast cancer cells via hTERT inhibition
Source: Sci Rep. 2024 Jan 22;14:1844. doi: 10.1038/s41598-024-51977-w (PMC10800350; doi:10.1038/s41598-024-51977-w)
Supplement: Supplementary file 1 — Supplementary Figures. [file 41598_2024_51977_MOESM1_ESM.pdf]

TAM	FAV	Effect %	CI
25	12.5	31.2	1.1
25	25	49.6	0.9
25	50	53.5	1.2
25	100	61.6	1.7
25	200	76.2	1.9
50	12.5	52.2	0.9
50	25	63.5	0.8
50	50	77.7	0.7
50	100	80.1	1.05
50	200	91.2	0.92

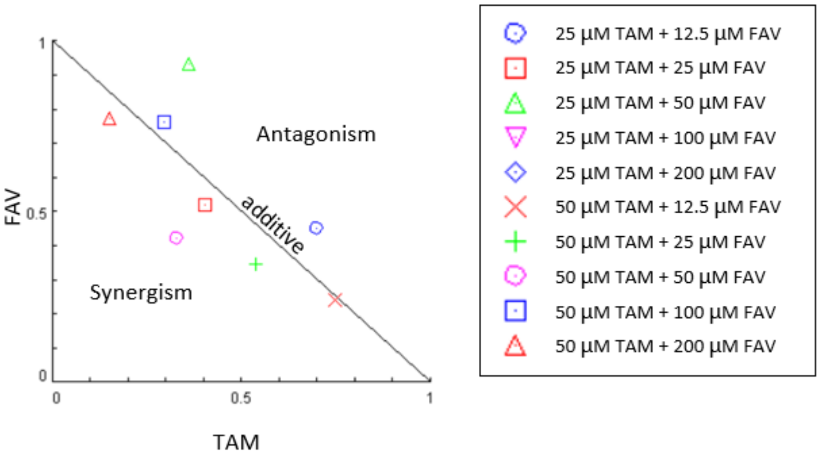

Fig. S1. Normalized isobologram of TAMR-1 cells. Data points below the line indicate synergistic effects, those on the line indicate additive effects, and those above the line indicate antagonistic effects. The data used in Compusyn was the average of three separate experiments performed in triplicate. CI; Combination index

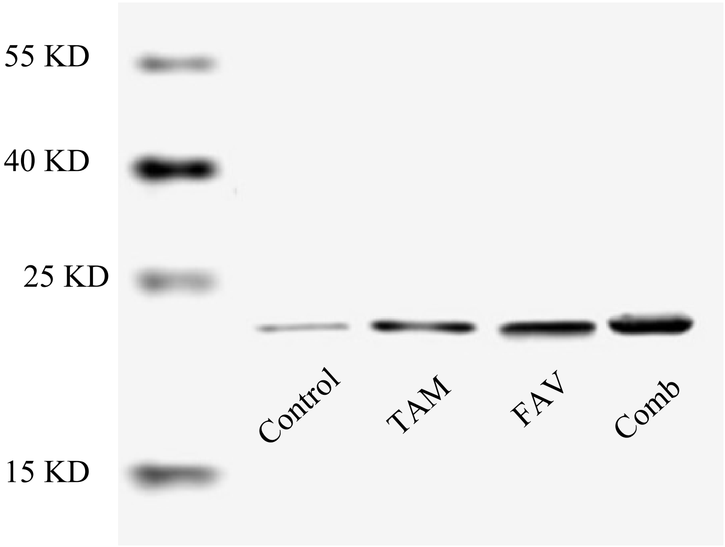

Fig.S2 Original western blot-BAX (23kD)

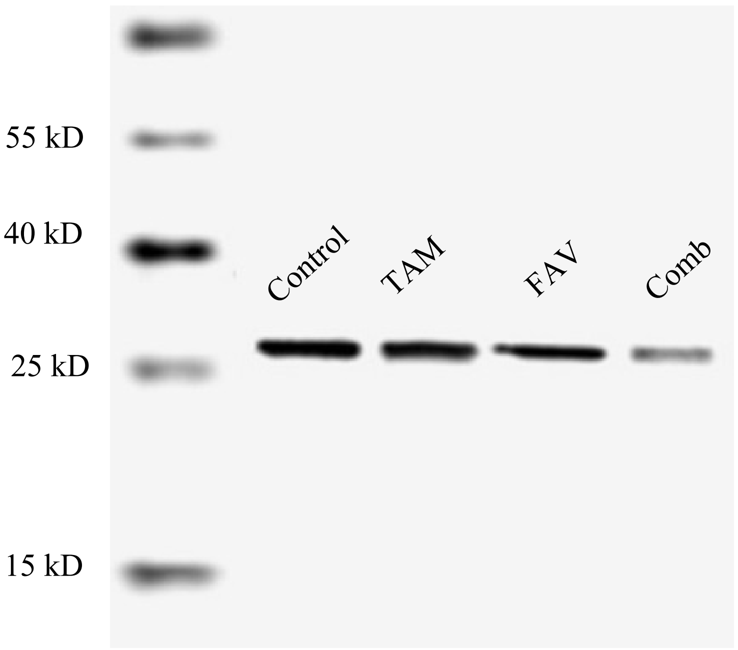

Fig.S2 Original western blot -BCL2 (26kD)

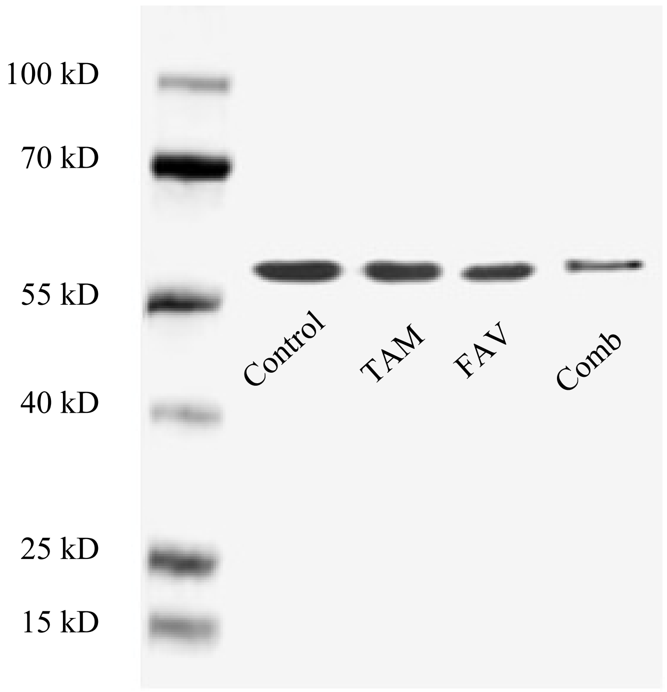

Fig. S3 Original western blot -CCNB1(60 kD)

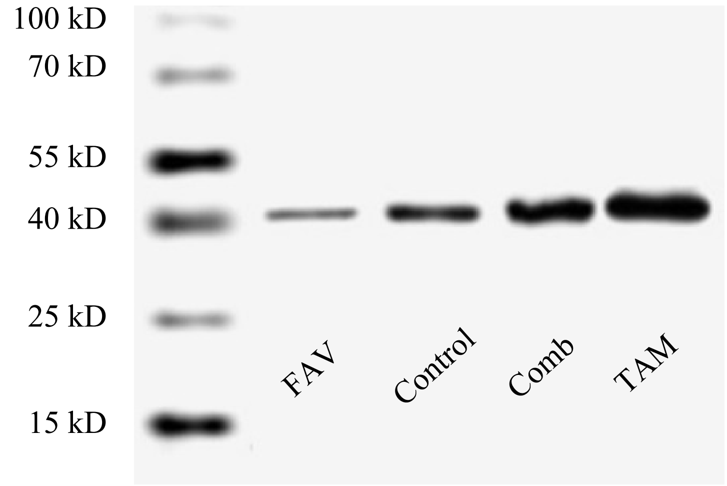

Fig.S3 Original western blot -CCND1(37kD)

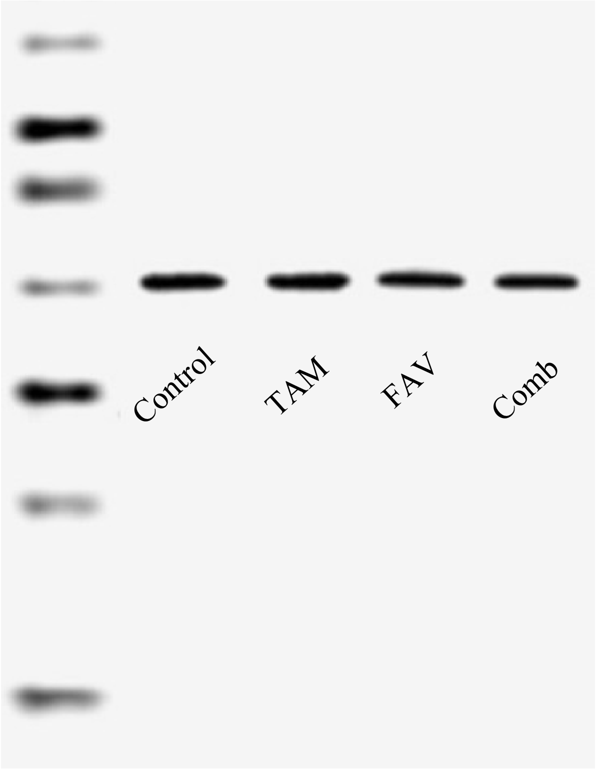

Fig.S2&3 Original western blot 1-β-actin (43kD)
